# A missense mutation in Lama3 causes androgen alopecia

**DOI:** 10.1038/s41598-023-48337-5

**Published:** 2023-11-27

**Authors:** Zhong-Hao Ji, Wen-Zhi Ren, Song He, Hong-Yu Wu, Bao Yuan, Jian Chen, Hong-Juan Jin

**Affiliations:** 1 Department of Plastic Surgery, The First Hospital of Jilin University, Changchun, 130062 Jilin China; 2 Department of Basic Medicine, Changzhi Medical College, Changzhi, 046000 Shanxi China; 3 Department of Laboratory Animals, College of Animal Sciences, Jilin University, Changchun, 130062 Jilin China; 4 Jilin Academy of Agricultural Sciences, Jilin City, 132101 Jilin China

**Keywords:** Genetic linkage study, Mutation

## Abstract

Hair loss disorders such as androgenetic alopecia have caused serious disturbances to normal human life. Animal models play an important role in exploring pathogenesis of disease and evaluating new therapies. NIH hairless mice are a spontaneous hairless mouse discovered and bred in our laboratory. In this study, we resequenced the genomes of NIH normal mice and NIH hairless mice and obtained 3,575,560 high-quality, plausible SNP loci and 995,475 InDels. The Euclidean distance algorithm was used to assess the association of SNP loci with the hairless phenotype, at a threshold of 0.62. Two regions of chromosome 18 having the highest association with the phenotype contained 345 genes with a total length of 13.98 Mb. The same algorithm was used to assess the association of InDels with the hairless phenotype at a threshold of 0.54 and revealed a region of 25.45 Mb in length, containing 518 genes. The mutation candidate gene Lama3 (NM_010680.2: c.652C>T; NP_034810.1: p. Arg217Cys) was selected based on the results of functional gene analysis and mutation prediction screening. Lama3 (R217C) mutant mice were further constructed using CRISPR/Cas9 technology, and the relationship between Lama3 point mutations and the hairless phenotype were clarified by phenotypic observation. The results showed that male Lama3 point mutation mice started to lose hair on the 80th day after birth, and the hair loss area gradually expanded over time. H&E staining of skin sections showed that the point mutation mice had increased sebaceous glands in the dermis and missing hair follicle structure (i.e., typical symptoms of androgenetic alopecia). This study is a good extension of the current body of knowledge about the function of Lama3, and the constructed Lama3 (R217C) mutant mice may be a good animal model for studying androgenetic alopecia.

## Introduction

Hair is an important accessory structure in the skin with physiological functions such as sensing stimuli, promoting damage repair, and immune regulation^1^. There are numerous human hair-related diseases, including alopecia, alopecia areata, and structural abnormalities of the hair shaft and hirsutism, which can cause serious disturbances to normal human life^2^. Androgenetic alopecia (AGA), is one of the most common hair loss disorders worldwide. AGA is a polygenic recessive disorder, but the gene for its pathogenesis has not been identified. AGA is highly heterogeneous in terms of severity, age of onset, and location of hair loss^3^. Currently, the main treatments for AGA include hair transplantation surgery and medication, which are expensive, have significant side effects and the therapeutic effect is very limited. Animal models play an important role in exploring the underlying mechanisms of disease development and evaluating new therapies.

The NIH hairless mouse (hereafter referred to as the HL mouse) is a spontaneous mutant mouse discovered in our laboratory during the breeding of NIH mice in 2007^4^. The HL mouse presents an autosomal recessive hairless phenotype from birth, can be accurately distinguished from normal mice at 7 days of age, and has normal thymus development. These characteristics are significantly different from those of the coat color mutant mice, indicating that HL mice are a novel type of hair mutant mouse. The breeding of animals and the localization of mutant genes provide important animal models and basic information for the study of the pathogenesis of hair loss, epidermal abnormalities and other skin-related diseases.

In this study, we used hairless and normal as extreme phenotypes and located the mutated gene on chromosome 18 by bulk segregant analysis (BSA). Based on the results of functional analysis and mutation prediction, we obtained the candidate gene Lama3, whose base 854 in exon 4 was mutated from C to T (NM_010680.2: c.652C>T; NP_034810.1: p.Arg217Cys). This is an unreported SNP locus that is completely linked to the phenotype. Laminin, a major component of the basement membrane, includes three isoforms, laminin-511, laminin-332, and laminin-211, and the Lama3 gene encodes the alpha subunit of laminin-332. Basement membrane-mediated molecular signaling between the epidermis and dermis is a major driver of skin development and hair dynamic homeostasis. Expression of laminin-511 and -332 changes dynamically with the hair growth cycle^5,6^. Supplementation with laminin-511 inhibits the transition from anagen stage VI to catagen of hair growth^7^, and also alleviates chemotherapy-induced hair loss^8^. And existing studies have shown that Laminin-511 deletion leads to defective hair papilla growth, which interferes with primary cilia formation and hair development^9^. Nonsense mutations in the Lama3 gene are a major cause of epidermal macrovesicular loosening^10,11^, and targeted disruption of the Lama3 gene in adult mice is sufficient to induce skin inflammation and fibrosis^12^. However, the function of the Lama3 gene in hair follicle morphogenesis and hair cycle transition remain to be elucidated.

Therefore, to clarify the relationship between the Lama3 (R217C) mutation and the hairless phenotype, this study used CRISPR/Cas9 technology to construct mutant mice with Lama3 (R217C) and observed the effect of the mutation on the skin and hair phenotype of mice at the animal and tissue levels. The results showed that Lama3 mutant mice started to show signs of androgenetic alopecia on the 80th day after birth. This study is a good extension of the current body of knowledge about the function of the Lama3 gene, and the constructed Lama3 single-base mutant mouse may be an animal model for the study of androgenetic alopecia.

## Materials and methods

### Laboratory animals

HL mice were bred at the Experimental Animal Center of Jilin University (SCXK (Ji) 2021-0001), and specific pathogen free (SPF)-grade NIH mice were purchased from Changchun Institute of Biological Products, Ltd. (SCXK (Ji) 2017-0005).

The experiments were conducted under the supervision of the Animal Ethics and Welfare Committee of Jilin University (approval number: SY201912015), all methods were carried out in accordance with relevant guidelines and regulations. This study was carried out in compliance with the ARRIVE guidelines, mice were anesthetized with isoflurane and euthanized by inhalation of excess carbon dioxide.

### Sample composition for bulked segregant analysis (BSA)

Two mixed pools were constructed for BSA with hairless and normal as extreme characteristics; the normal pool was named R03, and the hairless pool was named R04, and each pool contained DNA samples from 30 mice (15 males and 15 females) with the same amount of DNA in each sample.

### Preparation of DNA samples and genome resequencing

Liver DNA was extracted by the CTAB method, and after the samples were tested and quantified, DNA was randomly broken into 350 bp fragments by ultrasonic fragmentation, the DNA fragments were end-repaired, an A was added at the 3ʹ end, and sequencing connectors were added, purified, and PCR amplified to complete the construction of sequencing libraries. The libraries were sequenced by Illumina HiSeq after passing quality control. The raw image data files obtained from high-throughput sequencing were analyzed and transformed into raw sequenced sequences (sequenced reads), raw reads were converted to FASTAQ format, base identification (base calling) was performed by Illunima Casava 1.8 software, and clean reads were obtained after filtering. The short sequences obtained were compared with the reference genome using BWA software^13^, the positions of the clean reads on the reference genome were located by comparison, the sequencing depth and genome coverage of each sample was counted, and the variant sites were detected and statistically analyzed.

### SNP/InDel detection, filtering and result annotation

SNP/InDel detection was mainly implemented using the GATK software toolkit^14^. Based on the localization results of the clean reads to the reference genome, we used Picard software (http://sourceforge.net/projects/picard/) to mark duplicates and GATK for local realignment. Base preprocessing, such as base recalibration, was performed to ensure the accuracy of the detected SNPs/InDels, and then GATK was used to detect the SNPs/InDels and obtain the final SNP/InDel locus set. Subsequently, the SNPs/InDels were filtered to select high-quality loci for subsequent association analysis. The filtering criteria were as follows: first, SNP loci with multiple genotypes were filtered out; second, SNP loci with read support less than 4 were filtered out; third, SNP loci with consistent genotypes between the mixed pools were filtered out; and finally, high-quality SNP loci were obtained. Before using the InDels for association analysis, InDels was first filtered with the same filtering criteria as used for SNP analysis, and finally, high-quality, plausible InDel loci were obtained.

### Association analysis of hairless phenotypes with SNPs/InDels

In this study, the Euclidean distance (ED) algorithm was utilized, which is a method that uses sequencing data to find significantly different markers between the hybrid pools and subsequently uses this information to assess the region associated with the trait^15^. Theoretically, the ED value of non-target loci should tend to be 0. The formula of the ED method is shown below, and the larger the ED value, the greater the difference in the marker between the two pools.

$$ ED = \sqrt {\left( {A_{mut} - A_{wt} } \right)^{2} + \left( {C_{mut} - C_{wt} } \right)^{2} + \left( {G_{mut} - G_{wt} } \right)^{2} + \left( {T_{mut} - T_{wt} } \right)^{2} } . $$

When conducting the analysis, SNPs and InDels with differences in genotypes between the two mixed pools were used to count the depth of each base in the two pools and to calculate the ED value of each locus. To eliminate background noise, the original ED value was multiplied by a constant^15^, and in this project, to achieve the function of eliminating background noise, the 5th power of the original ED was taken as the association value, and then the ED value was fitted using the DISTANCE method.

### Enrichment analysis of genes within candidate regions

BLAST^16^ software was applied to perform deep annotation of genes within the candidate region in multiple databases. GO^17^, KEGG^18^ and COG^19^ enrichment analyses were performed for genes within the candidate region.

### Generation of Lama3 (R217C)-knockin mice using the CRISPR/Cas9 editing system

The mouse Lama3 gene (GenBank accession number: NM_010680.2; Ensembl: ENSMUSG00000024421) is located on mouse chromosome 18. Seventy-five exons have been identified, with the ATG start codon in exon 1 and TAA stop codon in exon 75. R217C is located on exon 4; therefore, Exon 4 was selected as the target site (sequences shown on the next page). A gRNA targeting vector and donor oligo (with a targeting sequence, flanked by a combined 138 bp homologous sequence on both sides) were designed. The p. R217C (CGT to TGT) mutation sites in the donor oligo was introduced into exon 4 by homology-directed repair. Two silent mutations (TCC to AGT, CTG to TTA) were also introduced to prevent the binding and cleavage of the sequence by gRNA after homology-directed repair. Cas9 mRNA and gRNA generated by in vitro transcription and donor oligo, were coinjected into fertilized eggs for KI mouse production. The pups were genotyped by PCR followed by sequence analysis. The gRNA (matching the reverse strand of the gene) was ATTACCTCGCCATTTTCCAGAGG.

Donor oligo:
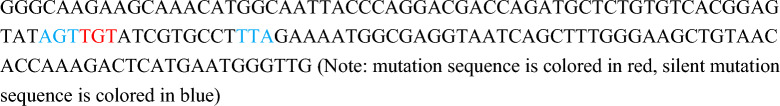


PCR Primers (Annealing Temperature 60.0 °C):

- Forward primer (F1): 5ʹ-TGCTTACTTGAGACATGAAGAG-3ʹ.
- Reverse primer (R1): 5ʹ-GTCCTGGATAGAGCTACTTGAG-3ʹ.

PCR product size 746 bp:

- Sequencing Primer: SF1: 5ʹ-TTGTCCTAAGCCTTCACCTAG-3ʹ.

### Photographing skin appearance and H&E staining of skin sections

Skin appearance was photographed at different time points, and the skin of the male Lama3 mutant mice and the corresponding control skin was cut at P150 from the dorsal hair loss area, fixed in 4% paraformaldehyde for 24 h, embedded in paraffin, and sectioned for H&E staining. The skin was subsequently observed and photographed by light microscopy (Olympus, Tokyo, Japan).

### ELISA

Testosterone and Dihydrotestosterone levels in serum were measured according to the operating instructions of an ELISA kit (mlbio, Shanghai, China).

### Statistical analysis

The experimental data are presented as the mean ± standard deviation. Unpaired t tests were used for analysis of differences between the two groups, and Welch’s test was used to correct for P values when F > 0.05. The data were analyzed and plotted using GraphPad Prism8 (Manufacturer, La Jolla, CA, USA). P < 0.05 was considered to indicate a significant difference.

### Ethics approval and consent to participate

The experiments were conducted under the supervision of the Animal Ethics and Welfare Committee of Jilin University (SY201912015), all methods were carried out in accordance with relevant guidelines and regulations. This study was carried out in compliance with the ARRIVE guidelines.

## Results

### Analysis of the genetic pattern of the hairless phenotype in HL mice

**Figure 1:**
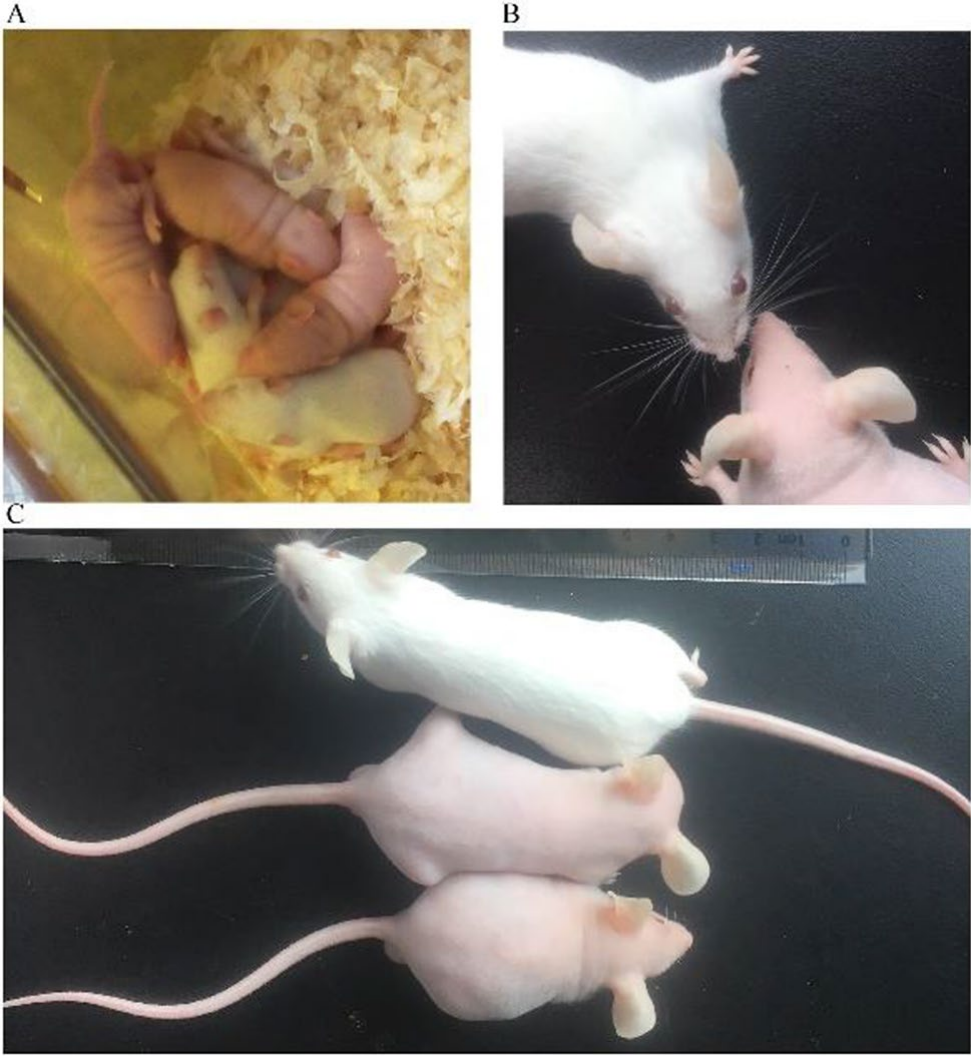
Phenotype of HL mice. (**A**) Appearance of HL mice and NIH mice on the seventh day after birth (P7). (**B**) Whiskers of HL mice and NIH mice at P50. (**C**) Appearance of HL mice and NIH mice at P50.

NIH mice were completely covered with hair on the body surface on the 7th day after birth (P7), while HL mice remained completely naked (Fig. 1A). In addition, HL mice had no whiskers (Fig. 1B) and still had no hair growth by the end of the second anagen (P50) (Fig. 1C). To clarify the inheritance pattern of the hairless phenotype in HL mice, this study set up various mating methods, such as test cross, self-cross, positive crossing and negative crossing, and deduced the inheritance pattern by observing the phenotypic segregation ratio of offspring.

The results in Table 1 show that all the offspring produced by the mating of the NIH mice F0 generation were normal, that all the offspring of HL mice had hairless phenotypes, that there was no difference in the results of forward and backward crosses, and that all the offspring of NIH and HL crosses had normal phenotypes, without the appearance of intermediate phenotypes. Based on the above analysis, the corresponding functional genes in the F0 generation of NIH and HL mice are pure, and the hairless phenotype can be inherited stably and independent of sex. These results suggest that the hairless mutation is an autosomal recessive mutation.

**Table 1:** Phenotype of F1 generation mice.

F0 generation parental composition (♂ × ♀)	Mating method	Number of hairless phenotypes of F1 generation	Number of normal phenotypes of F1 generation	Phenotype separation ratio (hairless: normal)
NIH × NIH	Self-cross	0	32	0
HL × HL	Self-cross	28	0	1
NIH × HL	Positive crossing	0	47	0
HL × NIH	Negative crossing	0	53	0

The results in Table 2 show that the offspring of the F1 generation of pure HL mice mated with heterozygous NIH mice showed phenotypic segregation, and the ratio was close to 1. The F1 generation of pure NIH mice mated with heterozygous NIH mice produced all F2 generations with normal phenotypes, proving that the hairless phenotype is a recessive trait. The F2 generations produced by self-crossing of the F1 generation of heterozygous NIH mice showed segregation of hairless and normal phenotypes.

**Table 2:** Phenotype of F2 generation mice.

F1 generation parental composition (♂ × ♀)	Mating method	Number of hairless phenotypes of F2 generation	Number of normal phenotypes of F2 generation	Phenotype separation ratio (hairless: normal)
HL (HL × HL) × NIH (HL × NIH)	Test cross	41	45	0.95
HL (HL × HL) × NIH (NIH × HL)	Test cross	37	49	
NIH (HL × NIH) × HL (HL × HL)	Test cross + negative crossing	24	19	
NIH (NIH × HL) × HL (HL × HL)	Test cross + negative crossing	42	38	
NIH (NIH × HL) × NIH (NIH × NIH)	–	0	20	0
NIH (NIH × HL) × NIH (NIH × HL)	Self-cross	17	62	0.29
NIH (HL × NIH) × NIH (HL × NIH)	Self-cross + negative crossing	24	80	

### Annotation and filtering of SNPs and InDels

The evaluation results of the sample base distribution ratio, insertion fragment distribution, and the base coverage and coverage depth proved that the library from the sequencing data was properly constructed with good randomness that covered the genome evenly (Fig. S1). The sequencing output data for the two sets of samples are shown in Table S1, and the Q30 values of the sequencing data are above 85%. The comparison results from the sequencing data with the reference genome are shown in Table S2, and the average comparison efficiency of both sets of samples was above 95%. The average coverage depths of the two sets of samples and the corresponding genome coverage ratios at each depth are shown in Table S3.

Based on the localization results for the samples on the reference genome from the clean reads, the SNPs and InDels with between sample differences were analyzed and summarized. The statistical results of SNPs and InDel between samples are shown in Fig. S2. The SNPs were filtered according to the following principles: SNPs with multiple genotypes, SNPs with read support less than 4, and then SNPs with genotypic concordance between the mixed pools. A total of 3,575,560 high-quality SNPs were obtained. The filtering criteria for InDels were the same as those for SNPs, and 995,475 high-quality, plausible InDel loci were finally obtained.

### Association analysis for the SNPs, InDels and hairless phenotype

**Figure 2:**
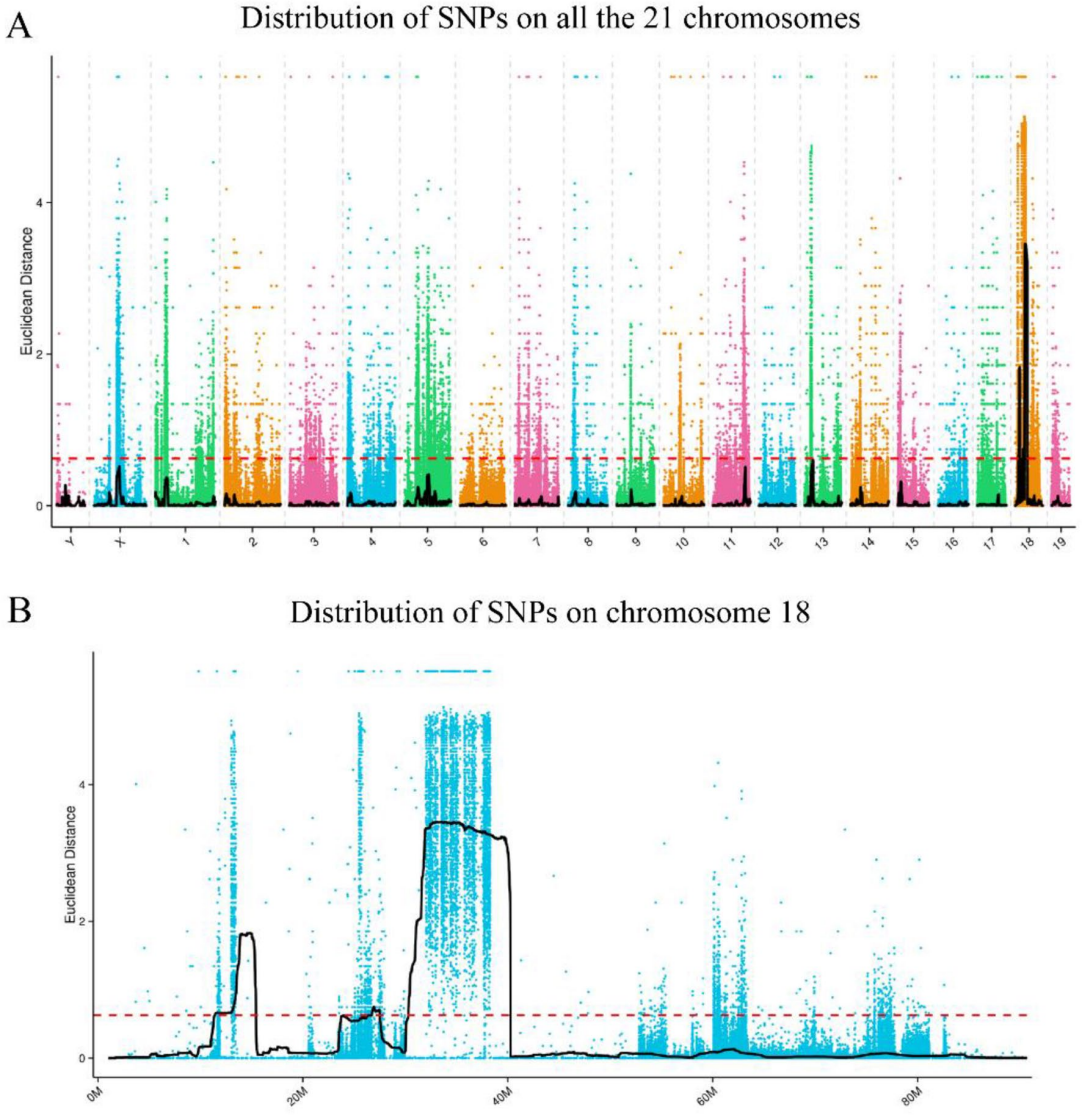
Results of SNP association analysis. (**A**) Distribution of SNP ED association values on all 21 chromosomes. (**B**) Distribution of SNP ED association values on chromosome 18.

The association of SNP loci with phenotypes was assessed according to the Euclidean distance (ED) algorithm. In this study, to eliminate background noise, the 5th power of the original ED was taken as the association value, and then the ED values were fitted using the DISTANCE method. The distribution of association values in all chromosomes is shown in Fig. 2A.

Some regions of chromosome 18 had the highest degree of correlation with the phenotype (Fig. 2B). Using 0.62 as the association threshold for analysis, the total length of the association region obtained was 13.98 Mb and contained 345 genes, with the specifics being shown in Table S4.

**Figure 3:**
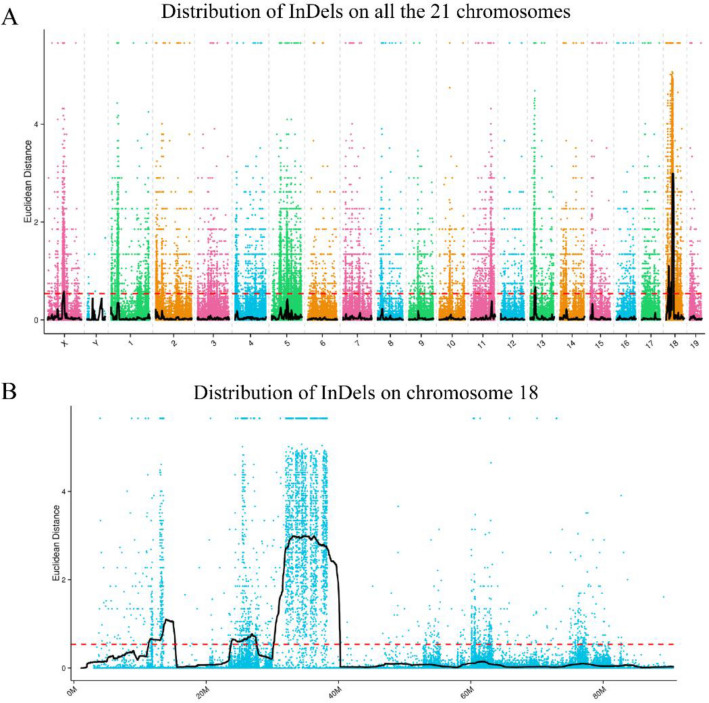
InDel association analysis results. (**A**) Distribution of InDel ED association values on all 21 chromosomes. (**B**) Distribution of InDel ED association values on chromosome 18.

The same algorithm assessed the association of InDels with traits, and the distribution of association values across all chromosomes is shown in Fig. 3A, with the highest degree of association between some regions of chromosome 18 and the phenotype (Fig. 3B).

The analysis was performed with an association threshold of 0.54, and six regions with a total length of 25.45 Mb containing 518 genes were obtained, and the specifics are shown in Table S5. Furthermore, we show the results of the association analysis for the SNPs (Fig. S3A) and InDels (Fig. S3B) by circle plots. From outside to inside, the order is as follows: the first circle is chromosome coordinates; the second circle is gene distributions; the third circle is SNP and InDel density distributions; and the fourth circle is the distribution of ED values corresponding to the SNPs and InDels. The detailed results of the SNP and InDel association analysis are shown in Tables S6 and S7.

### Gene enrichment analysis of candidate regions

The region with the highest correlation with the phenotype on chromosome 18 was selected as the candidate region, and BLAST software was applied to deeply annotate the coding genes in this region with multiple databases (NR, Swiss-Prot, GO, KEGG, COG). A total of 309 genes were annotated in the candidate region, among which a total of 41 genes were annotated with nonsynonymous mutations between the mixed pools and 2 genes were annotated with code-shifting mutations. The annotation results are shown in Table S8. The results of the enrichment analysis of GO, COG and KEGG are shown in Fig. S4A–C, respectively. Among them, KEGG analysis results showed that the Hippo signaling pathway, PI3K-Akt signaling pathway, MAPK signaling pathway and the neuroactive signaling pathway were enriched.

### Lama3 c.652C>T(p. R217C) mice show an androgenetic alopecia-like phenotype

**Figure 4:**
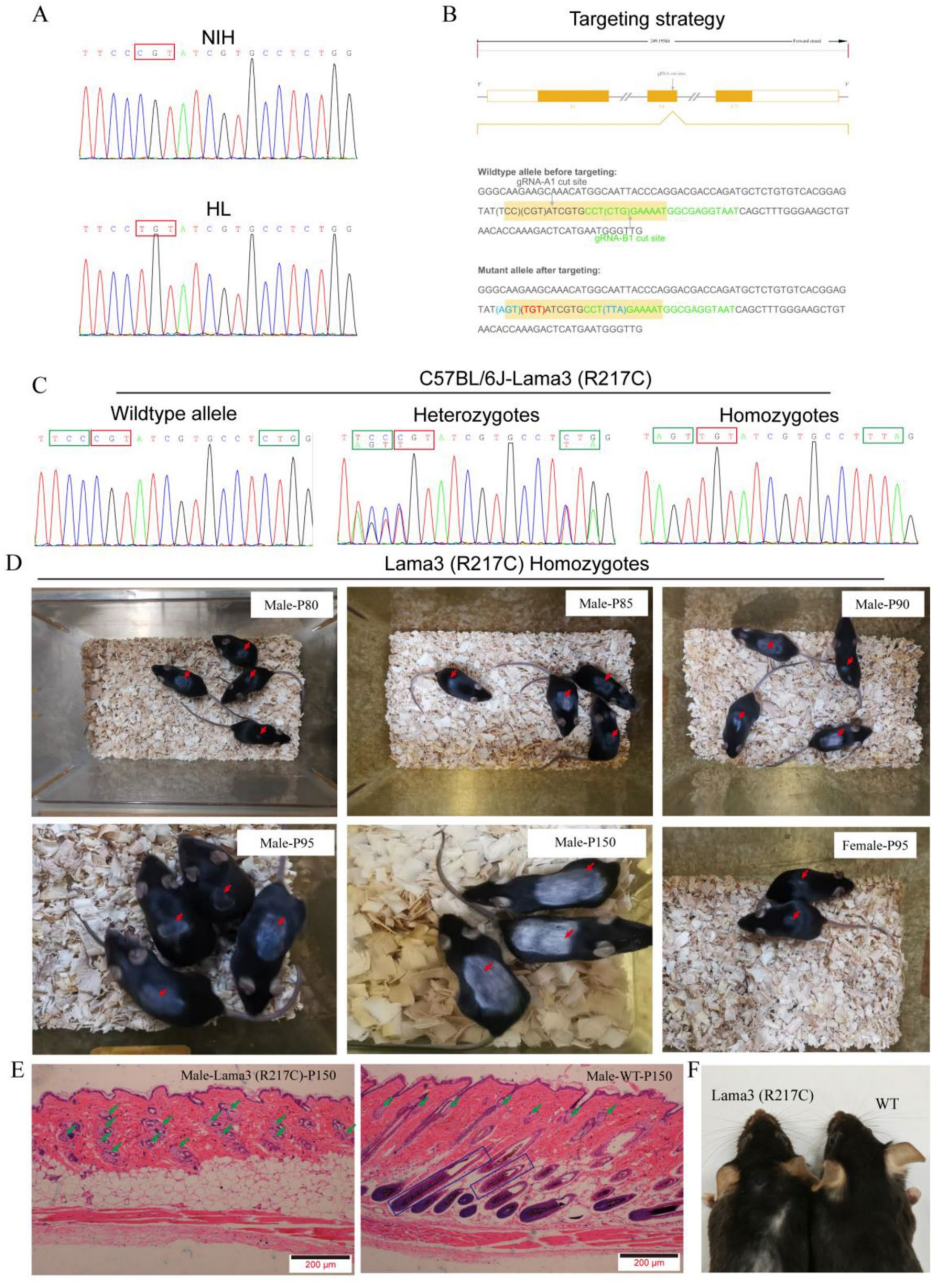
Preparation and phenotypic analysis of Lama3 (R217C) mutant mice. (**A**) PCR+ Sanger sequencing to identify HL mice with C to T mutations in the Lama3 gene. (**B**) Schematic diagram of Lama3 mutant mice constructed by CRISPR-Cas9 technology. Red markers are the mutation sites, and green markers are the two introduced synonymous mutations. (**C**) PCR+ Sanger sequencing to identify the genotype of F2 generation mice. (**D**) Skin phenotype results of male Lama3 mutant mice on P80, P85, P90, p95, and P150 and female Lama3 mutant mice on P95. The red arrows in the figure mark the area of hair loss. (**E**) H&E staining results of skin sections of male Lama3 mutant mice on P150, with sebaceous glands marked by green arrows and hair follicle structures marked by rectangular boxes in the figure. (**F**) Magnified image of whiskers in Lama3 (R217C) point mutant mice and wild-type (WT) mice.

The mutation candidate gene Lama3 (NM_010680.2: c.652C>T; NP_034810.1: p. Arg217Cys) was selected based on the results of gene function analysis and mutation prediction screening. PCR amplification followed by Sanger sequencing verified that the missense mutation was indeed present in Lama3 in HL mice (Fig. 4A). To clarify the effect of the Lama3 (R217C) mutation on the hair phenotype, we constructed point mutation mice with Lama3 by CRISPR/Cas9 technology, and the construction strategy is shown in Fig. 4B where the red markers are mutation sites, and the green markers are two synonymous mutations introduced with the aim of reducing off-target effects. Heterozygotes were mated to obtain pure heterozygotes, and the mutation sites were verified by Sanger sequencing (Fig. 4C). The Lama3 (R217C) mutation had no significant effect on the biological properties of mice such as body size, growth and organs (results not shown). The hair phenotypes of the mice were observed, and the results showed that the Lama3 (R217C) mutant mice behaved normally in the first two hair growth cycles, while male mice from the anagen phase of the third hair growth cycle (P80) showed hair loss starting from the center of the back with the area of hair loss gradually expanding over time and finally forming a regional baldness with clear boundaries (P150). In contrast, female mice showed inconspicuous spot hair loss at P95 (Fig. 4D). However, areas of clearly defined baldness similar to those in male mice are also formed at P150. H&E staining of skin sections showed that male wild-type C57BL/6J mice (WT-P150) had an intact skin structure with normal sebaceous gland distribution and hair follicles extending down into the subcutaneous tissue, whereas male Lama3 (R217C) had absent hair follicles and significantly more sebaceous glands in the dermis compared to controls (Fig. 4E). The whiskers of Lama3 (R217C) point mutant mice have no obvious abnormalities and are very similar to those of wild-type (WT) mice (Fig. 4F). Meanwhile, we examined the serum levels of androgens in Lama3 R217C mutant mice, and the results showed that the levels of testosterone and dihydrotestosterone in male mutant mice were not significantly different from those in wild-type mice (Fig. S5A,B), whereas the levels of testosterone and dihydrotestosterone were significantly elevated in female mutant mice (P < 0.01) (Fig. S5C,D). In conclusion, the phenotypic analysis showed that male Lama3 point mutation mice exhibit typical AGA features.

## Discussion

Hair mutation mouse models have a wide range of applications in life science and medical research, such as skin photoaging damage^20–22^ and parasitic infections^23^, and are an important part of animal models for human diseases. Hairless (Hr)^24,25^, Gsdma3^26,27^, and Foxn1^28^ are several hair-related functional genes reported in the study. Their nonsense mutations or missense mutations cause hairlessness or progressive hair loss. However, animal models related to hair abnormalities are still scarce. We also have very limited knowledge of hair function genes.

In the present study, we used normal and hairless as extreme phenotypes and association analysis of variant loci and phenotypes by the BSA technique, and the mutated genes were initially localized to chromosome 18. Lama3, an extracellular ligand molecule of the PI3K/Akt signaling pathway, can activate the PI3K pathway by binding to integrin receptors on the membrane^29^. It has been shown that the PI3K/Akt signaling pathway is essential for the morphogenesis of hair follicles. The PI3K-Akt pathway inhibitor treatment in hair follicle reconstruction experiments results in failure of follicle regeneration^30^. Additionally, our previous transcriptome sequencing results showed that the PI3K-Akt pathway was in a repressed state in HL mouse skin^4^. Therefore, we initially selected the Lama3 gene as a mutation candidate. Furthermore, we constructed Lama3 (R217C) mutant mice by CRISPR/Cas9 technology, and the observation of the hair phenotype showed a causal relationship between the Lama3 (R217C) mutation and AGA.

AGA is a common polygenic disorder whose genetics are not fully understood. Several studies have revealed the association of genetic SNPs with AGA and patchy baldness^31–33^. For example, a study by Seok et al. showed that the HSPA1B SNP rs6457452 was associated with patchy baldness in a Korean population^34^, and a study by Antonio Prodi et al. showed that the EDAR SNP rs1352015 was associated with AGA^35^. These studies analyzed the correlation of these SNPs with hair loss disorders such as AGA, but the causal relationship between the two remains uncertain.

Lama3 (R217C) is a missense mutation newly identified by us, and the site is highly conserved among species, corresponding to the human LAMA3 protein R220C. In the present study, following a classical forward genetics approach, we formed a complete logical closure loop from the new hairless phenotype discovered and performed mutant gene screening for the subsequent verification of the causal relationship between the gene and the phenotype. We demonstrated for the first time that the Lama3 (R217C) mutation causes AGA. This locus has screening and diagnostic value for AGA.

Of course, there are limitations to our study, and there are many meaningful questions that still need to be examined. First, the mutation of Lama3 (R217C) did not fully recapitulate the hairless phenotype of HL mice, which suggests that the combination of multiple genes may be behind the hairless phenotype of HL mice; however, the whole-genome resequencing results from the HL mice still have great value for excavation and exploration. Second, we established a causal link between Lama3 (R217C) mutations and AGA, but the underlying molecular mechanisms remain unresolved. In this regard, we also went through additional steps. First, we found through bioinformatics analysis that the mutation of Lama3 (R217C) might lead to the loss of ADP-ribosylation modification at this site, which in turn may have affected the binding of the Lama3 ligand to the integrin receptor on the cell membrane and led to the inhibition of the downstream PI3K-AKT pathway. However, the lack of Lama3 IP-level commercial antibodies and its extra-long CDS region (9993 bp) limited our experiments. We may subsequently analyze the effect of point mutations on their ability to bind to the receptor by constructing truncated bodies containing labeled antibodies. Third, the question remains whether the mutant locus is present in patients with clinical AGA. Establishing the answer to this question directly determines the clinical significance and subsequent application value of the locus.

In conclusion, in this study, we performed resequencing analysis of HL mice, screened for SNPs and InDels with linkage to the phenotype, and further constructed Lama3 (R217C) mutant mice by CRISPR/Cas9 technology to clarify the causal relationship between this point mutation and AGA. Lama3 (R217C) mutant mice may be an important model for studying the pathogenesis of AGA, and this locus has potential as a diagnostic and screening test for AGA.

### Supplementary Information

Supplementary Information.

## Data Availability

The raw sequence data reported in this paper have been deposited in the Genome Sequence Archive in National Genomics Data Center, China National Center for Bioinformation/Beijing Institute of Genomics, Chinese Academy of Sciences (GSA: CRA009211) that are publicly accessible at https://ngdc.cncb.ac.cn/gsa/browse/CRA009211.

## Abbreviations

AGA: Androgenetic alopecia
BSA: Bulk segregant analysis
CDS: Coding sequence
ED: Euclidean Distance
H&E: Hematoxylin and eosin
InDel: Insertion-deletion
SNP: Single nucleotide polymorphism
SPF: Specific pathogen free
