# Supplementary material for: A missense mutation in Lama3 causes androgen alopecia
Source: Sci Rep. 2023 Nov 27;13:20818. doi: 10.1038/s41598-023-48337-5 (PMC10682005; doi:10.1038/s41598-023-48337-5)
Supplement: Supplementary file 1 — Supplementary Information. [file 41598_2023_48337_MOESM1_ESM.zip › Supplementary figures.docx]

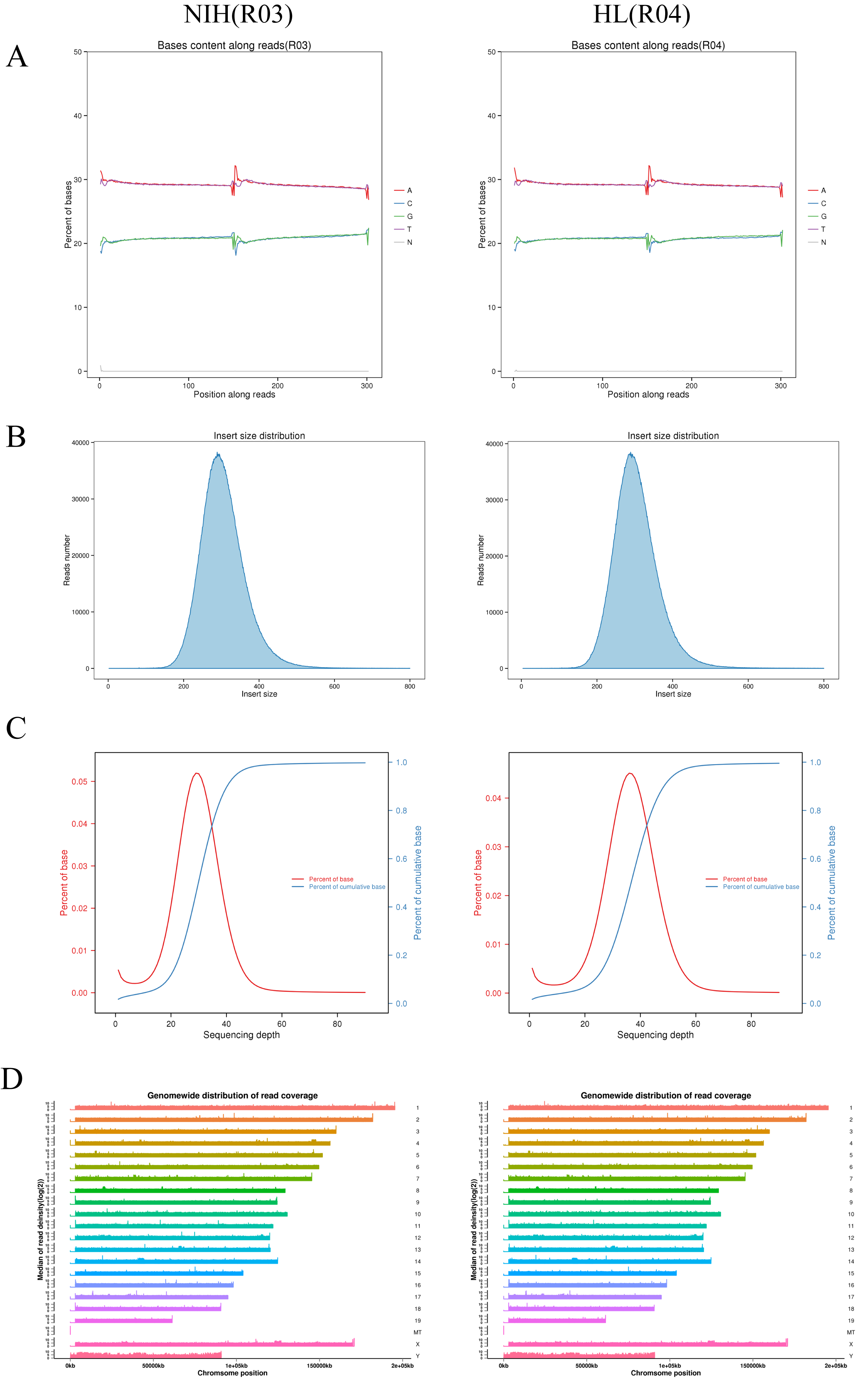


Figure S1. Sequencing data quality control

A: Distribution of bases in two groups of samples.

Green represents base G, blue represents base C, red represents base A, purple represents base T, and gray represents base N which is not identified in sequencing.

B: Distribution of insert fragments.

C: Distribution curves of base coverage depth and coverage distribution of samples.

The horizontal coordinate is the sequencing depth, the left vertical coordinate is the percentage of bases corresponding to that depth, corresponding to the red curve, and the right vertical coordinate is the percentage of bases at that depth and below, corresponding to the blue curve.

D: Distribution of chromosome coverage depths of the two sets of samples.


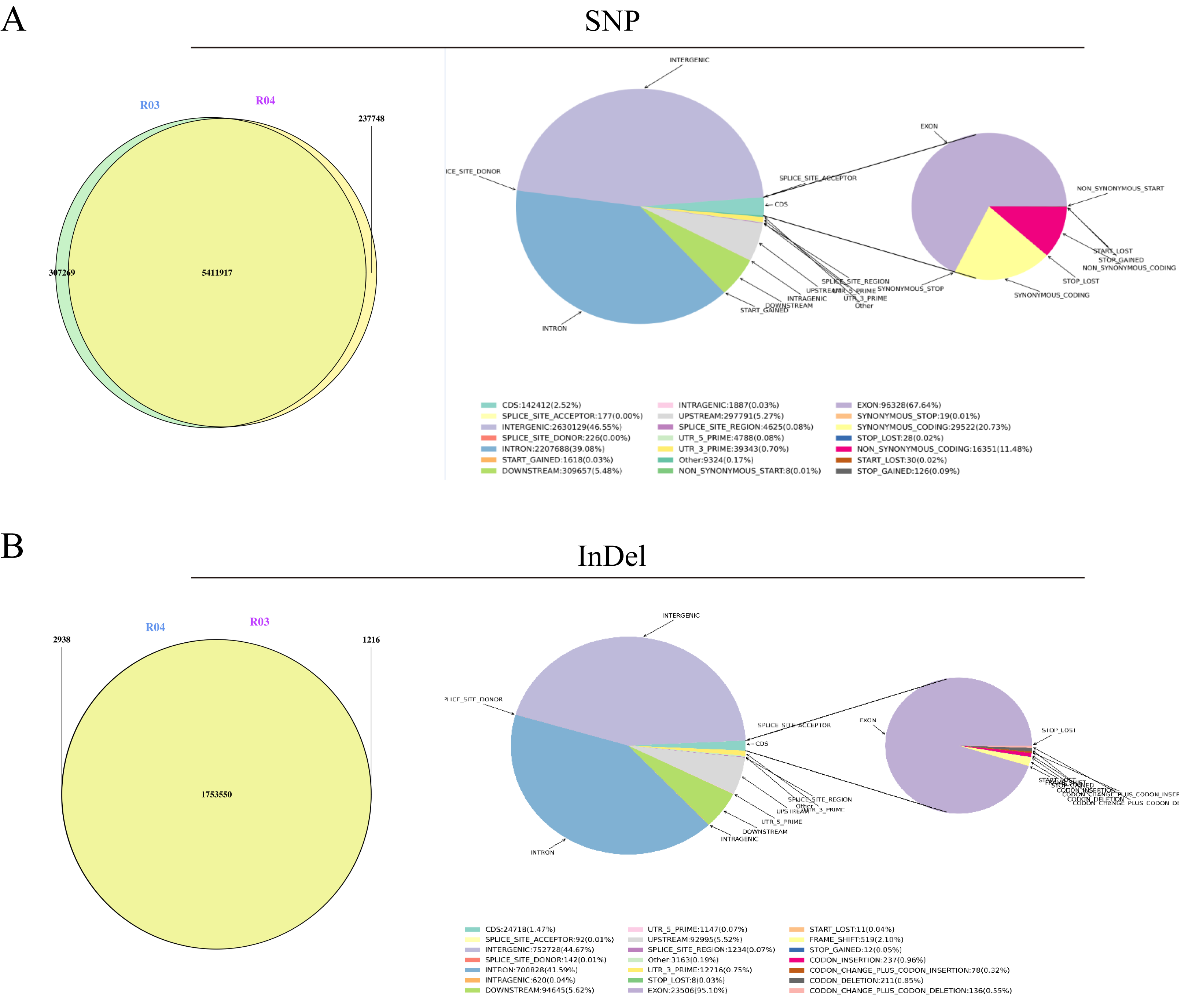


Figure S2. Annotation of differential SNPs and InDels between mixed pools

A: Venn diagram of SNP statistics between samples and annotation results of SNPs in HL mouse samples at varying genomic positions.

B: Venn diagram of InDel statistics between samples and the annotation results of InDels in HL mouse samples at varying genomic positions.


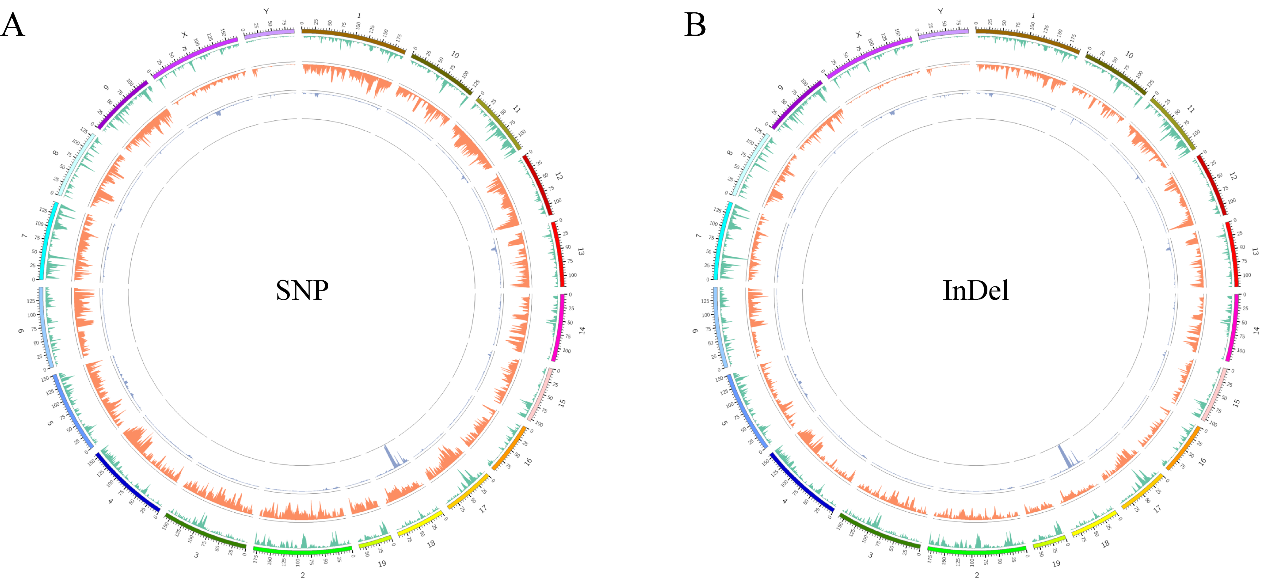


Figure S3. Visualization of SNP and InDel analysis results

A: Circle diagram showing the SNP analysis results.

B: Circle diagram showing the results of the InDel analysis.

Note: From outside to inside, the first circle are the chromosome coordinates; the second circle are the gene distributions; the third circle are the SNP and InDel density distributions; and the fourth circle are SNPs and InDels corresponding to the ED value distribution.


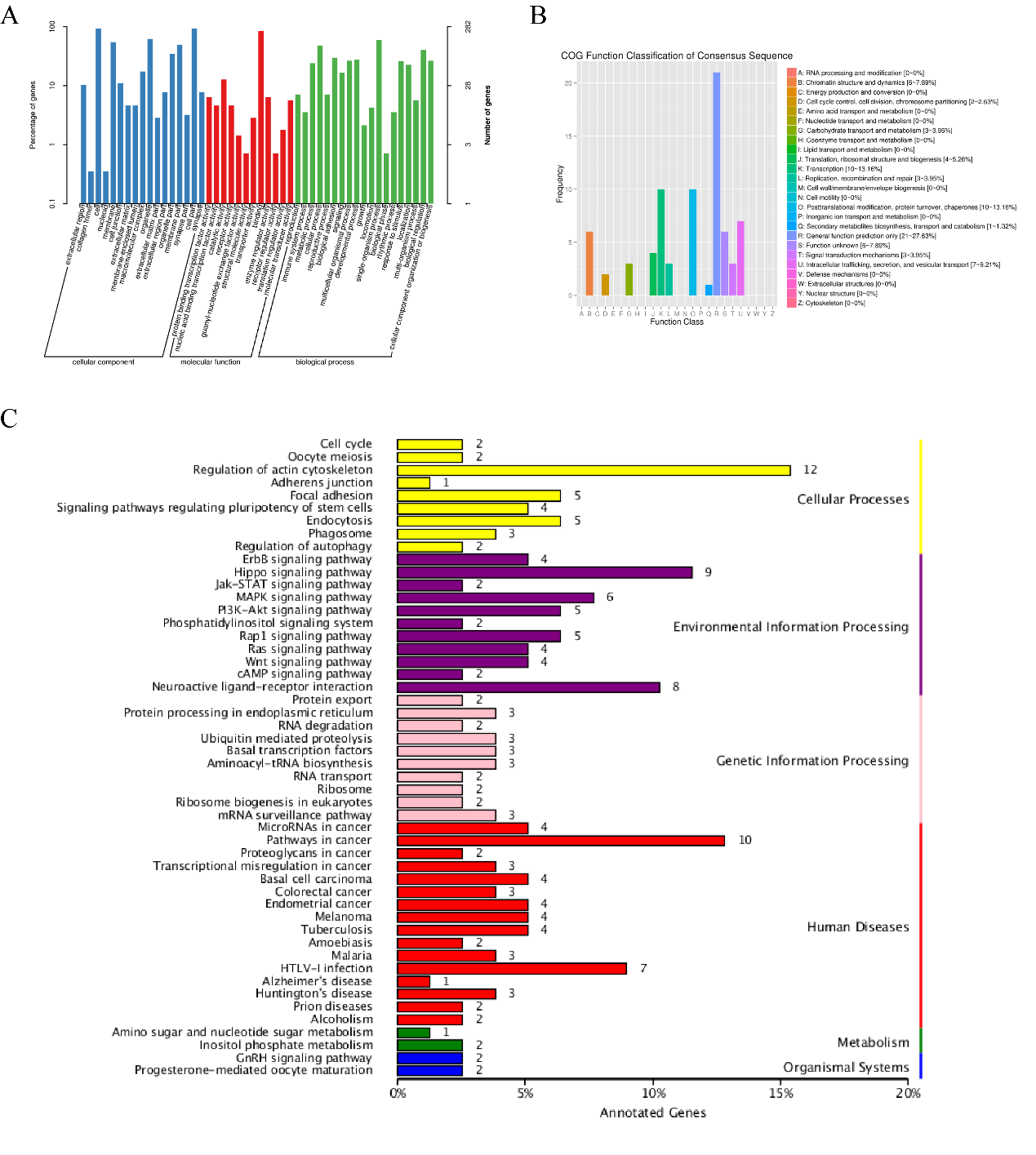


Figure S4. Enrichment analysis results of genes in candidate regions

A: Results of GO enrichment analysis.

B: COG enrichment analysis results.

C: Results of KEGG enrichment analysis.


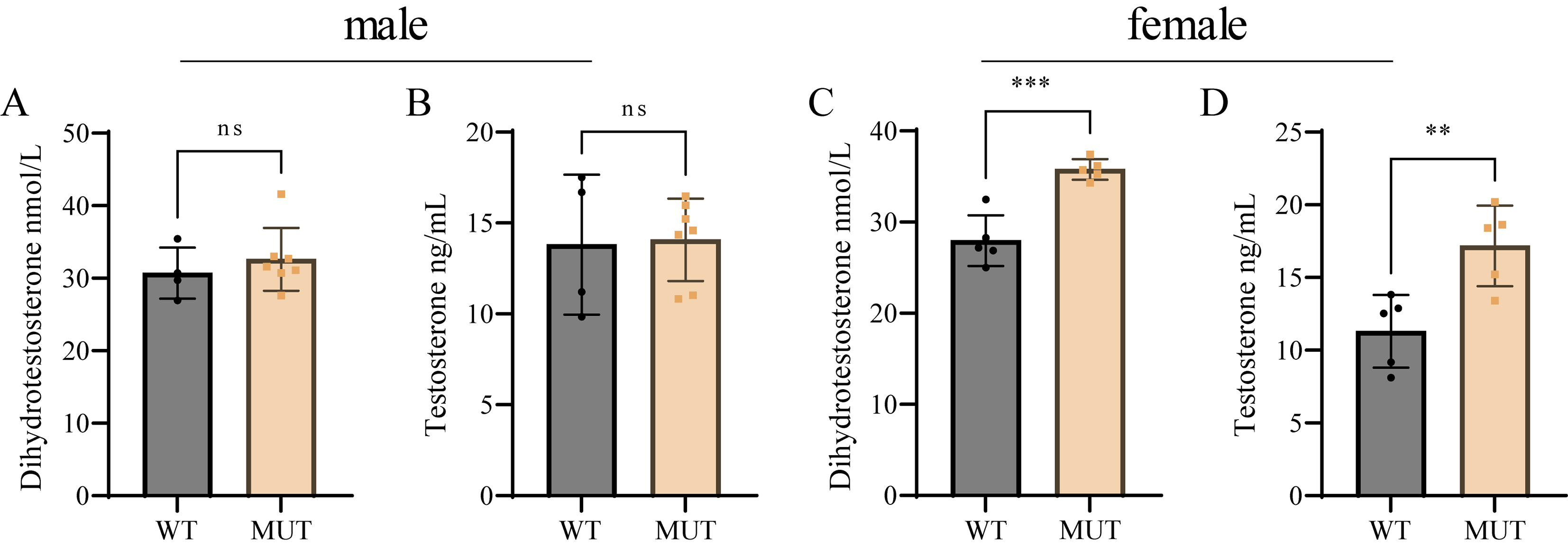


Figure S5. Detection of serum androgen levels using ELISA

A: Serum levels of dihydrotestosterone in male mice.(WT n=4; MUT n=7)

B: Serum levels of testosterone in male mice.(WT n=4; MUT n=7)

C: Levels of dihydrotestosterone in serum of female mice.(WT n=5; MUT n=5)

D: Levels of testosterone in the serum of female mice.(WT n=5; MUT n=5)

Note: WT indicates wild-type mice, MUT indicates Lama3 R217C mutant mice, ** *P* < 0.01, *** *P* < 0.001.
